# Therapeutics update in immune-mediated rheumatic diseases: Rheumatoid arthritis, idiopathic inflammatory myositis and ANCA-associated vasculitis

**DOI:** 10.1016/j.clinme.2025.100507

**Published:** 2025-09-02

**Authors:** Caroline Zollinger-Read, Andrew Filer

**Affiliations:** a Rheumatology Research Group, Department of Inflammation and Ageing, College of Medicine & Health, University of Birmingham, Birmingham, UK; b National Institute for Health and Care Research (NIHR) Birmingham Biomedical Research Centre, University Hospitals Birmingham NHS Foundation Trust, Birmingham, UK

**Keywords:** Biologics, Rheumatoid arthritis, ANCA associated vasculitis, Idiopathic inflammatory myositis

## Abstract

Immune-mediated inflammatory diseases (IMIDs) are a group of common clinically diverse conditions that are characterised by systemic inflammation. They often pose medical challenges due to their multi-organ involvement, chronicity, associated comorbidities and poor impact on quality of life for patients.

The management for IMIDs has changed profoundly over the past 20 years, with the paradigm of treatment shifting away from broad immunosuppression towards pathway-specific targeted treatment. This editorial will focus on three immune mediated rheumatic diseases that physicians may encounter in their clinical practice; rheumatoid arthritis (RA), idiopathic inflammatory myositis (IIM) and anti-neutrophil cytoplasm antibodies (ANCA)-associated vasculitis (AAV). This editorial will provide a concise overview of the current targeted treatment landscape of these three conditions.

## Highlights

• Treatment of RA, IIM and AAV focuses on early recognition, targeted immunosuppression and limiting toxicity.
• Myositis specific antibodies have improved prognostic stratification and disease classification of idiopathic inflammatory myositis.
• MDA-5 is associated with rapidly progressive ILD and TIF1γ is related to malignancy.
• Avacopan should be considered in severe active AAV.
• Avacopan reduces glucocorticoid related morbidity.

## Rheumatoid arthritis

Rheumatoid arthritis (RA) is a chronic multisystem autoimmune disease that has a high disability rate through persistent inflammation and ultimately irreversible damage to joints. The aim of treatment for RA is to achieve remission and prevent disease progression, which is best achieved by a treat-to-target approach. Joint damage in RA is caused by inflammation of the synovium, known as synovitis, which is driven by a number of pro-inflammatory cytokines, such as tumour necrosis factor alpha (TNF-α), interleukin 6 (IL-6) and interleukin 1 (IL-1).^1^

The treatment of RA has evolved immensely over recent decades since the development of biological disease-modifying anti-rheumatic drugs (bDMARDs, known as biologics), and targeted synthetic disease-modifying anti-rheumatic drugs (tsDMARDs). Biologics are monoclonal antibodies or related genetically engineered molecules that target specific aspects of pathogenesis in RA, such as B cells, T cells and cytokines. In the UK, the National Institute of Health and Care Excellence (NICE) approves biologics use in patients with RA who have active disease and failed to respond to two or more conventional disease-modifying anti-rheumatic drugs (cDMARDS).^2^

The first biologic for RA was infliximab, which became available in 2000. Infliximab inhibits the activity of the cytokine TNF-α. There are now five TNF inhibitors: adalimumab, etanercept, infliximab, golimumab and certolizumab. TNF inhibitors were the cornerstones of treatment for RA, but now other biologics with different modes of actions have been developed, including IL-6 inhibitors (eg tocilizumab), rituximab (CD-20 monoclonal antibody) and abatacept (co-stimulatory molecule inhibitor), summarised in Table 1.^3^ IL-1 inhibitors (anakinra) were previously used in RA; however, these are no longer used due to reduced efficacy compared with other targeted therapies.

**Table 1:** Current bDMARDs and tsDMARDs available for patients with RA.

Medication	Mechanism of action	Administration and dosage
Adalimumab^a^	Humanised monoclonal antibody to TNF-α receptor; inhibits TNF-α binding to its receptor	Subcutaneous (SC) injection, 50 mg every other week
Etanercept^a^	Cloned fusion protein of human TNF-α receptor 2 and inhibits TNF-α binding to its receptors	SC injection, 50 mg weekly
Infliximab^a^	Chimeric monoclonal antibody TNF-α receptor; inhibits TNF-α binding to its receptor	Intra-venous (IV) infusion, 3 mg/kg every 4 weeks, dose can be increased to maximum of 7.5 mg/kg every 8 weeks
Golimumab^a^	Humanised monoclonal antibody to TNF-α receptor; inhibits TNF-α binding to its receptor	SC injection, 50 mg every 4 weeks (can be increased dose to 100 mg if >100 kg or not responding)
Certolizumab^a^	PEGylated Fab' fragment of a humanised monoclonal antibody to TNF-α receptor; inhibits TNF-α binding to its receptor	SC injection, 400 mg initially then 200 mg every other week
Rituximab	Chimeric monoclonal antibody to CD20 antigen on B-cell lymphocytes	IV infusion, two 1,000 mg infusions 2 weeks apart every 6 months based on clinical assessment
Abatacept	Soluble fusion protein which blocks co-stimulatory signal mediated by the CD28-CD80/86 pathway, required for T-cell activation	IV infusion, <60 kg 500 mg every 4 weeks, 60–100 kg 750 mg every 4 weeks, >100 kg 1 g every 4 weeks or SC injection, 125 mg weekly
Tocilizumab	Humanised monoclonal antibody that binds and inhibits interleukin-6 receptor	IV infusion, 8 mg/kg every 4 weeks or SC injection, 162 mg weekly
Sarilumab	Humanised monoclonal antibody that binds and inhibits interleukin-6 receptor	SC injection, 200 mg every 2 weeks
Filgotinib	Selective inhibitor of JAK-1 and JAK-2	Tablet, 200 mg daily (100 mg in older people)
Baricitinib	Selective inhibitor of JAK-1 and JAK-2	Tablet, 4 mg daily (2 mg in older people)
Tofacitinib	Selective inhibitor of JAK-1 and JAK-3	Tablet, 5 mg twice daily
Upadacitinib	Selective inhibitor of JAK-1	Tablet, 15 mg daily

a BSR 2023 pregnancy guidelines advise that all TNF inhibitors are compatible in pregnancys.^6^

Recent years have seen the focus shift to the newest class of therapeutic agent, JAK inhibitors, which are small molecule oral tsDMARDs. JAK inhibitors have multiple modes of actions; they interrupt the JAK-STAT pathway, a crucial signalling cascade that regulates immunity.^4^ There are four JAK inhibitors approved for RA; filgotinib, baricitinib, tofacitinib and upadacitinib. Their oral administration, rapid onset and ability to be used as monotherapy offer compelling advantages compared to biologics. Clinical trials have nuanced our understanding of JAK inhibitors and their adverse events, in particular the risk of thromboembolic and cardiovascular events in high-risk patients.^5^ In 2023, the European Medicines Agency safety committee advised risk minimisation measures when prescribing JAK inhibitors to avoid in patients aged 65 years or older, current or previous smoker, malignancy or other cardiovascular risk factors.

The choice of biologic or tsDMARD is therefore dependent on patient comorbidities, patient preferences, cost and careful consideration of safety profiles. Previously, for patients with RA to be eligible for biologics, a high disease activity score was required. However, in 2021 NICE made all TNF inhibitors, filgotinib and upadacitinib available to patients with moderate disease activity for RA.

## Idiopathic inflammatory myositis

Idiopathic inflammatory myositis (IIM) represents a heterogeneous group of diseases characterised by immune-mediated muscle damage, often accompanied by other organ involvement including skin, lungs or joints. IIM is associated with increased interstitial lung disease (ILD) and malignancy risk, which is accompanied by marked morbidity and mortality.^7^, ^8^, ^9^

IIMs pose unique diagnostic and therapeutic challenges due to their rarity, heterogenous clinical presentation and limited therapeutic options. Over the past decade, our understanding of the pathogenesis of IIM has advanced with the critical discovery of myositis-specific antibodies. These antibodies have refined IIM classification by delineating subtypes of myositis into three main groups; dermatomyositis, antisynthetase syndrome and immune-mediated necrotising myopathy.^10^ Approximately 70% of adults with IIM have an identifiable myositis autoantibody.^11^ The ability to stratify patients by autoantibody profile has allowed for more precise diagnosis, prognosis and a personalised approach to management of IIM.

The presence of autoantibody anti-melanoma differentiation-associated gene 5 (MDA-5) has been correlated with rapidly progressive ILD, especially in East Asian populations, and these patients have a high mortality rate.^7^ One in four patients with IIM will develop cancer within 3 years of their diagnosis, particularly individuals with the myositis-specific autoantibodies such as transcriptional intermediary factor 1-gamma (anti-TIF1γ) and anti-nuclear matrix protein 2 (anti-NXP2).^7^ More than 50% of patients with anti-TIF1γ will have an underlying cancer and these patients need an enhanced cancer screening.^9^ The International Myositis Assessment and Clinical Studies Group has published a guideline for IIM-associated cancer screening, which stratifies patients into standard, moderate and high risk groups based on their myositis subtype, autoantibody status and clinical features. In addition, the guideline also recommends the appropriate cancer screening investigations based on their risk profile.^8^

Glucocorticoids remain the first-line therapy in IIM with typical doses of 0.5–1 mg/kg per day, usually 40–60 mg is recommended. In acute and severe cases of IIM, intravenous high-dose pulses of methylprednisolone up to 1 g per day for 3–5 days are used. Maintenance therapy is commenced in parallel with glucocorticoids; commonly used cDMARDS include azathioprine, methotrexate, mycophenolate and tacrolimus. In refractory or severe disease, stronger immunosuppression such as rituximab and cyclophosphamide should be considered.^12^ Rituximab can be used in severe IIM when conventional therapy has failed, while abatacept can be considered as an alternative treatment in patients with severe IIM.^13^ Intravenous immunoglobulins are a second-line treatment in patients with active IIM with dysphagia or refractory skin disease.^14^ In 2023, the British Society for Rheumatology (BSR) published an evidence-based guideline on the management of patients with IIM.^8^

There has been emerging promising data in the management of IIM observed with JAK inhibitors including tofacitinib, baricitinib, ruxolitinib and upadacitinib.^15^ Preliminary data from a recent randomised control trial demonstrated that treatment of IIM patients with baricitinib resulted in improved clinical response after 24 weeks.^16^ Current evidence for JAK inhibitors in IIM is limited to case reports and a small number of clinical trials. Therefore, larger clinical studies are required to ascertain the therapeutic efficacy in this patient population.

## ANCA-associated vasculitis

ANCA-associated vasculitis (AAV) represents a spectrum of systemic small vessel vasculitides and can present with life-threatening manifestations, such as diffuse alveolar haemorrhage and rapidly progressive glomerulonephritis.^17^ AAV is divided into three distinct clinical-pathological phenotypes including granulomatosis with polyangiitis (GPA), microscopic polyangiitis (MPA) and eosinophilic granulomatosis with polyangiitis (EGPA), and are associated with proteinase 3 (PR3) and myeloperoxidase (MPO) antibodies. AAV commonly affects the upper airways, lungs, kidneys, skin, eyes and peripheral nervous system, although any organ can be affected. Diagnosing AAV can be challenging due to its varied clinical manifestations and overlapping features with other autoimmune conditions.^17^

Therapeutic advances have revolutionised the management of AAV. Once managed almost exclusively with high-dose glucocorticoids and cyclophosphamide, current immunosuppressive regimes emphasise remission induction with biologics and lowest possible glucocorticoid exposure. The BSR updated their AAV management clinical guidelines in 2025, based on latest available evidence.^17^ For new-onset active AAV (GPA or MPA), induction treatment should include high-dose glucocorticoids, typically three pulses of intravenous methylprednisolone or prednisolone with starting doses of 50–75 mg or 1.0 mg/kg/day, alongside immunosuppression with rituximab or cyclophosphamide. Concurrent rituximab and cyclophosphamide use can be considered for organ- or life-threatening disease. Rituximab is the preferred treatment for relapsed GPA or MPA, rather than cyclophosphamide, due to fewer long-term complications.^17^ In relapsing-refractory EGPA, mepolizumab (a monoclonal antibody that targets interleukin 5) is recommended for induction therapy.^18^ Plasma exchange can be used as an adjunctive therapy in GPA or MPA for patients with severe renal involvement with a serum creatinine >300 µmol/L; however, plasma exchange is not recommended in diffuse alveolar haemorrhage.^17^ The PEXIVAS trial demonstrated that plasma exchange reduced the risk of end-stage kidney disease in patients presenting with severe AAV. For new-onset AAV, oral glucocorticoids (prednisolone) should be tapered in accordance with the PEXIVAS trial tapering schedule, which aims to reach a dose of 5 mg prednisolone by 4 months.^19^

The most recent advance in AAV therapy has been the introduction of avacopan treatment following the ADVOCATE trial. Avacopan is an oral C5A receptor antagonist, now recommended as an adjunctive steroid-sparing agent for severe active AAV.^17^, ^18^, ^19^, ^20^ In ADVOCATE, glucocorticoid-related adverse effects were significantly lower in the avacopan-treated group, in addition to a more rapid reduction in proteinuria and improved renal recovery.^21^ Avacopan is an attractive option for patients at high risk of glucocorticoid-related morbidity such as diabetes, osteoporosis and infections. The recent BSR AAV guidelines have a strong emphasis on how steroid dose should be rapidly tapered to prevent long-term steroid toxicity in AAV. Although current AAV therapies have extended the survival of patients, 50% of AAV patients will relapse: therefore, AAV patients need careful specialist long-term monitoring.

## Conclusion

Treatment for RA has evolved dramatically over the last 20 years, with a plethora of biologics and targeted therapies now available. In the field of IIM, the greatest advance has been the landmark discovery of myositis-specific antibodies that allow IIM-associated cancer risk stratification at presentation. Well-designed controlled clinical trials are required to assess the role of targeted therapies in IIM and develop a more evidence-based approach to treatment. In AAV treatment, there has been a shift to rapid tapering of steroids in combination with avacopan to minimise steroid-related toxicity. In a subgroup of AAV patients with active relapsing disease, rituximab is the preferred induction agent. Treatment for RA, IIM and AAV all now share the same goal: to provide sustained remission, improve quality of life, reduced risk of relapses and minimise treatment-related side effects.

## CRediT authorship contribution statement

**Caroline Zollinger-Read:** Writing – original draft, Project administration, Conceptualization. **Andrew Filer:** Writing – review & editing.

## Declaration of competing interest

Professor Andrew Filer reports a relationship with Johnson & Johnson Innovative Medicine that includes: consulting or advisory and funding grants. Professor Andrew Filer reports a relationship with Quell Therapeutics Limited that includes: consulting or advisory. Professor Andrew Filer reports a relationship with Sonoma Pharmaceuticals that includes: consulting or advisory. Professor Andrew Filer reports a relationship with Bristol Myers Squibb Co that includes: funding grants. Professor Andrew Filer reports a relationship with Roche that includes: funding grants. Professor Andrew Filer reports a relationship with Nascient that includes: funding grants. Professor Andrew Filer reports a relationship with Mestag Therapeutics Limited that includes: funding grants. Professor Andrew Filer reports a relationship with GSK that includes: funding grants. Professor Andrew Filer reports a relationship with Synact Pharma that includes: funding grants. If there are other authors, they declare that they have no known competing financial interests or personal relationships that could have appeared to influence the work reported in this paper.
